# Sanicula Spring Water

**Published:** 1890-09

**Authors:** J. G. Gundlach

**Affiliations:** Spokane Falls, State of Washington


### SANICULA SPRING WATER.

EDITORS OF THE HOMŒOPATHIC PHYSICIAN:—Your number for June is at hand, and while each monthly visit is looked forward to with interest and satisfaction, I must say the remarks of Dr. H. C. Morrow on Sanicula were a very great source of satisfaction to me, even a delight. As Dr. S. Lilenthal said in reference to the case of constipation cure by Sanicula, as reported by Prof. J. T. Kent in the *Medical Advance* of January, 1889, each and every clinical verification is so much proof that our work is so far well done and that all our suffering has not been in vain. I doubt very much whether any remedy has been proven to the extent of Sanicula, nor will any one undertake it again after what has been placed on record. In this case the daily use of the water extended over one year, which accounts for its long action, and though some five years since the proving was made, we all (that is, my family) still suffer from the effects, and I fear never will fully get over them, as most all the symptoms still recur. The fact that Calc. had failed to help Dr. Morrow, and that Sanicula did, shows how much deeper is its action. We all know how characteristic this condition is of Calc., and yet it failed. Will Dr. Berridge have the kindness to report in full the cured case in THE HOMŒOPATHIC PHYSICIAN for May? It is in this way alone that a remedy can be developed and made useful after proving. I gave a number of similar cases to I. H. A., 1889, which I trust may help to bring the Sanicula into the prominence it deserves. Should Dr. Lee have to get out a supplement to his *Repertory*, I hope he will remember Sanicula. Why not get some kind of a binder that could be used to put the installments in as they come out? All would buy them, I am sure.

Yours,

J. G. GUNDLACH, M. D.

SPOKANE FALLS, STATE OF WASHINGTON.

